# Whole-Transcriptome Selection and Evaluation of Internal Reference Genes for Expression Analysis in Protocorm Development of *Dendrobium officinale* Kimura *et* Migo

**DOI:** 10.1371/journal.pone.0163478

**Published:** 2016-11-04

**Authors:** Hongqiang An, Qiankun Zhu, Wei Pei, Jing Fan, Yi Liang, Yihui Cui, Nan Lv, Wanjun Wang

**Affiliations:** School of Life Science and Engineering, Southwest Jiaotong University, Chengdu 610031, the People’s Republic of China; Wuhan University, CHINA

## Abstract

*Dendrobium officinale* Kimu *et* Migo has increased many researchers’ interest for its high medical and horticultural values and the molecular mechanism of its protocorm development remains unclear. In this study, 19 genes from 26 most stably expressed genes in whole transcriptome of protocorms and 5 housekeeping genes were used as candidate reference genes and screened with 4 application softwares (geNorm, NormFinder, BestKeeper and RefFinder). The results showed that a few reference genes could effectively normalize expression level of specific genes in protocorm development and the optimal top 2 reference genes were *ASS* and *APH1L*. Meanwhile, validation of *GNOM*, *AP2* and temperature induced gene (*TIL*) for normalization demonstrates the usefulness of the validated candidate reference genes. The expression profiles of these genes varied under protocorms and temperature stress according to the stablest and unstablest reference genes, which proved the importance of the choice of appropriate reference genes. The first systematic evaluation of stably expressed genes will be very useful in the future analysis of specific genes expression in *D*. *officinale*.

## Introduction

Gene expression analysis is fundamentally important for identifying genes relevant to biological processes and provides insights into complex regulatory networks in which they are involved [1]. Quantitative real-time reverse transcription-PCR (RT-qPCR) [2] is one of the most reliable and reproducible techniques to measure and evaluate gene expression level [3] in order to confirm or interpretate the gene expression profiles [4]. Meanwhile, RT-qPCR results are inevitably affected by different experimental conditions, such as different amounts of starting materials, quality and integrity of template RNA samples, primer design and reverse transcription efficiency [5]. Additionally, random pipetting errors can also add technical variability to the data [6,7]. These factors can potentially render the quantification of gene transcripts unreliable. In order to avoid non-specific variations or errors, it is essential to select stably expressed genes as internal standard to normalize the expression levels of target genes in unknown samples. These stably expressed genes are often called endogenous control or internal reference genes (IRGs).

Ideal IRGs should be stably expressed at different developmental stages and in various tissues. They should not be influenced by experimental procedures or co-regulated with the target genes. They should also be expressed in relatively abundance and have minimal innate variability [8]. Traditionally, IRGs have been chosen from housekeeping genes [9,10]. The most frequently used ones in expression studies include *Actin* (*ACT*), *glyceraldehyde-3-phosphate dehydrogenase* (*GAPDH*), *Tubulin* (*TUB*), 18S rRNA gene, *Ubiquitin* (*UBQ*), and *translation elongation factor 1-α* (*EF1α*) [11,12], etc. Unfortunately, there is no universal IRGs that is expressed at a constant level under all conditions or in all tissues [12,13], and different species may have different available IRGs. The indiscriminate use of some housekeeping genes as IRGs is questionable, since their expression levels may be regulated according to different conditions [14–16]. Therefore, it is essential and fundamental to select appropriate IRGs for normalization of expression levels of target genes in appropriate biological process.

*Dendrobium officinale* Kimu *et* Migo, a well-known orchid plant in some countries of South and Southeast Asia, is valuable for its attractive flowers and medical use. Their embryogenesisis at a standstill at globular stage and the mature seed consisted of an immature globular embryo packaged by testa [17,18]. The mature seed can germinate to form a yellowish green globular cell mass which called protocorm [17,18]. Analogic globular cell mass, which is called protocorm-like body (PLB), can also be formed through somatic embryogenesis [19,20]. Protocorms or PLBs are specific structures during the process of seed germination or somatic embryogenesis in orchids. According to our recent study, asymbiotic germination of *D*. *officinale* seeds pass through various stages including embryo activation (EA), protocorm (PC), promeristem (PM), shoot apical meristem (SAM), spheroidicity protocorm (SP), leaf primordium and vascular system (LPVS), root apical meristem (RAM), degeneration of protocorm (DP), etc (data not published). However, a series of molecular mechanism underlying the process remains unknown. *D*. *officinale* will be a model to investigate the molecular mechanism of the specific embryo development in orchids. We found some genes that are specifically expressed in the protocorms of *D*. *officinale*, in which a *NAC* gene, *DcNAC*, is specifically expressed in the promeristem [20]. In order to understand the regulatory mechanism of *DcNAC*s and find more specific genes underlying the developmental process of protocorms, we carried out transcriptome sequencing of three samples at stages of protocorm, promeristem, SAM, respectively. A plethora of annotated unigenes relevant to plant development are found and many of them showed differential expressions. In order to identify the key genes involved in the developmental process, one of the prerequisites is to understand the expression profile. Therefore, it is essential to select and evaluate IRGs for expression analysis of protocorm development in *D*. *officinale*. So far, there hasn't been any comprehensive report of IRGs from protocorm or PLB development. In this study, 31tested genes from annotated transcriptomic unigenes of protocorms were used to find IRGs for gene expression stability analysis during protocorm development in *D*. *officinale*.

## Materials and Methods

### 2.1 Plant materials and treatments

Protocorms of *D*. *officinale* were come from asymbiotically germinated seeds (harvested from the plants grown up on the pot in the laboratory) cultured on seed germination (SG) medium, in which the constituents are half macrocomponents, whole microcomponents, ferric salt components, and organic components of MS basal medium, supplemented with 3% sucrose and 0.6% agar, and pH was adjusted to 5.8 with 1 mol·L^-1^ NaOH or HCl. Plantlets originated from the protocorms were cultured on plantlet growth (PG) medium, supplemented with 1.07μM NAA on SG medium. The culturing chamber was set at 25±1°C and 14hrs lighting in each day.

Abiotic stress treatments (PEG6000 and temperature stress) were performed under dark environment using aseptic young plantlets with 3~4 leaves, which are 3±0.2cm tall. In PEG6000 stress treatment, the plantlets were cultured on PG medium supplemented with 16.67mM PEG6000 for 1hr, 6hr, 12hr, 24hr and 48hr. In temperature stress treatment, the plantlets were cultured on PG medium at 5°C in freezer and 35°C in incubator for 1hr, 6hr, 12hr, 24hr and 48hr, respectively.

### 2.2 Total RNA isolation and cDNA preparation

Protocorms (at 6 stages of EA+PC, PM, SAM+SP, LPVS, RAM, DP from asymbiotically germinated seeds, and PLBs from embryonic calli), tissues (roots, stems and leaves from aseptic young plantlets cultured on PG medium, seeds and whole blooming flowers from the plants grown up on the pot in the laboratory), stem parts (shoot tip, node and internode from aseptic young plantlets cultured on PG medium), and the stressed plantlets were collected respectively. Total RNAs were extracted using Plant RNA Kit (OMEGA BIO-TEK), which were treated with RNase-free DNase I (TaKaRa) to remove genomic DNA, and should be suitable for RT-qPCR studies according to their OD_260_/OD_280_ ratios and electrophoresis in 1% agarose gel. Concentration and purity of isolated total RNAs were calculated from OD_260_/OD_280_ with SYNERGY^H1^ microplate reader (BioTek^®^), the integrity checked by electrophoresis in 1% agarose gel.

Transcriptomic analysis of protocorms at 3 stages (PC, PM and SAM) was performed using Illumina HiSeq^™^2000by Biomarker Technologies Co., Ltd (Beijing) (data not published), Reverse transcriptionsof total RNAs were performed with 1μg of total RNA in a total volume of 20μl with 2μl of 50μM oligo-dT(18) primer and 0.5μl of 200U/μl Reverse Transcriptase M-MLV (RNase H^-^) (TAKARA) according to the manufacture’s recommendations, respectively. Before transcription, total RNAs and oligo-dT(18) primer were mixed and incubated at 70°C for 10min followed by cooling on ice more than 2min. The first strand cDNA synthesis was proceeded at 42°C for 1hr after adding M-MLV, dNTP mix, transcriptase buffer and RNase Inhibitor, followed by 70°C for 15min. All cDNA samples were diluted 1:10 with RNase-free water and then stored at -80°C freezer.

### 2.3 Primer design, RT-qPCR amplification and IRGs’ selection

26 unigenes, in which the difference of gene expression among protocorms at 3 stages was less than 3.5%, and 5 housekeeping genes (*Actin*, *Tubulin*, *EF-1α*, *GAPDH*, *SAND*), which are most commonly used as internal control for expression studies [12], were selected as tested genes in this study (Table 1 and S1 Table). Gene symbol, Nr-Annotation, primer sequences, amplicon length, Tm values, amplification efficiency and correlation coefficients were listed in Table 1. cDNAs of 31 tested genes were cloned with 3’RACE and 5’RACE technique according to their transcriptomic sequences and sequenced by Sangon Biotech (Shanghai) Co., Ltd. Primers for RT-qPCR amplification were designed according to the cDNA sequences of the tested genes using primer Premier 5.0 software with a melting temperature between 58–65°C, primer length 20–26bp, GC content about 50% and amplification lengths 100–300bp, and then synthesized by Sangon Biotech (Shanghai) Co., Ltd.

**Table 1:** Primer sequences and amplicon characteristics of 31 tested genes and 2 *Actin* genes of *D*.*officinale*.

No.^4^	Unigene No.	Gene Symbol	Annotation	Primer pairs used for RT-qPCR (forward / reverse primer)	S-T (%)^2^	Amplicon length (bp)^3^	Amplicon Tm (°C)	R±SE^1^	E±SE^1^
1	T2-29401	*GABAT3*	Probable gamma-aminobutyrate transaminase 3	GGGAGATAAGAGGCACAGGC / CTATCTCCAGCAACGCGGAT	0.71	151	83.95	0.9910±0.0060	1.9213±0.0425
2	T2-34503	*NMCP1L*	Putative nuclear matrix constituent protein 1-like protein	TGGAGCAACCAATGTTGGGA / ATTGCTCACCGGTCGACTAC	1.15	184	84.36	0.9987±0.0011	1.9687±0.0232
3	T2-26423	*MOS2*	Protein MOS2	AGATCGCCGCTGGATTCAAA / GTCAACCACCGAACTGGACT	1.60	193	82.82	0.9987±0.0012	2.0030±0.0300
4	T2-29783	*PAXIP1*	PAX-interacting protein 1	GTGGTAACGCCTATGTGGCT / AGCGAAACAGGCATGCTGAA	1.66	122	81.92	0.9984±0.0018	1.9470±0.0491
5	T3-26893	*HDAC5*	Histone deacetylase 5	ATTTGGGAGAGCAGGATGGC / TTGCAACAGCGTCCTTGAGA	1.69	90	83.08	0.9996±0.0002	1.9280±0.0375
6	T1-28366	*KU8*	ATP-dependent DNA helicase 2 subunit KU8	ATGGAGACAGGGACAAGGGA / CTGCCGTGATGCAGGTAGAT	1.69	163	80.92	0.9985±0.0020	1.9767±0.0784
7	T1-23605	*RPL30*	60S ribosomal protein L30	TTGTTTAGTGCCGACGACGA / CTCAGACTTGCGGAGTGGAG	1.90	249	85.05	0.9995±0.0002	1.9220±0.0165
8	T3-27882	*SFT2B*	Vesicle transport protein SFT2B	CGGGAGCACAGCATTCCTTA / GTACCAGACAAGGGCACCAA	2.26	187	82.37	0.9983±0.0024	1.9253±0.1204
9	T2-34737	*UBC24*	Probable ubiquitin-conjugating enzyme E2 24	TGGGTGGTGATGAACTGCAA / CACCCTCTTCAGCCAACCAT	2.35	240	83.47	0.9971±0.0032	1.9573±0.0242
10	T2-17479	*T2-17479*	Uncharacterized protein At2g34160	TACGTCAACCTCGCCAAGAG / AGCAGCCATCAGCTCATCAA	2.53	246	84.52	0.9980±0.0018	1.9710±0.0550
11	T3-12872	*TPRXL*	Putative protein TPRXL	TGTTCGACTTCGGTCAGCTC / GGGTACTGCTTTCTCGCACT	2.53	104	85.34	0.9990±0.0003	1.9593±0.0598
12	T3-20348	*CPSF5*	Cleavage and polyadenylation specificity factor subunit 5	TCCTCCACACATCACCAAGC / TCACTGGCCCATACCTCTGA	2.55	152	81.37	0.9991±0.0013	2.0007±0.0317
13	T3-17931	*TFIIB*	Transcription initiation factor IIB	CAAGCAGTCAAAGCTGCACA / GGCAAAGGAGGTCGGAATGA	2.60	231	82.96	0.9996±0.0002	1.9073±0.0129
14	T2-29412	*APH1L*	Gamma-secretasesubunit APH1-like	TGCCTGCGTACTTTGCATTG / GTACCAAAGGAGGGGCCAAA	2.78	181	83.09	0.9972±0.0012	1.9220±0.0218
15	T3-22550	*BIP1*	Luminal-binding protein 1	AGGAGTTCGCAGAGGAGGAT / CTTGAGAGCCGCATCGATCT	2.88	98	83.10	0.9973±0.0031	1.9513±0.0415
16	T3-13226	*GT3b*	Trihelix transcription factor GT-3b	AGTGATCAAGAACGGCCCAG / TCTCCCACCAATGGGACTCT	2.90	140	85.94	0.9992±0.0013	1.9983±0.0595
17	T3-11823	*T3-11823*	unknown	AATATCAGGCCTCCGCATCG / GCCCTGAAGCAGTGAGGAAT	2.90	103	82.60	0.9986±0.0017	1.9723±0.0565
18	T3-12831	*T3-12831*	Uncharacterized protein GN = PGSC0003DMG400021318	TGGCGATCACACCACAATGA / GTGCAGGCTCGTGACAACTA	3.01	135	81.83	0.9986±0.0018	1.9783±0.0530
19	T1-29649	*ASS*	Argininosuccinate synthase	GTGCTGACCGTTGATCCAGA / AGAAGCGGGAGAGAGTTCCT	3.03	111	84.32	0.9990±0.0007	1.9493±0.0339
20	T1-22498	*TCP1γ*	T-complex protein 1 subunit gamma	GCTGCTTCCATGTTGCTGAG / TCTGGAAGCATCTGCTCGT	3.03	138	84.01	0.9993±0.0006	2.0050±0.0408
21	T3-23608	*DLD*	Dihydrolipoyl dehydrogenase	TGCCTGGCTTTGATCCTGAG / GGCTGCATCTACCTCCAACA	3.08	188	81.85	0.9993±0.0006	1.9770±0.0757
22	T2-21987	*TXNL2*	Thioredoxin-like 2	GTTTCGTGGTTCATGCGGTC / TCACCCGCTTGGCTTAATGT	3.16	127	82.56	0.9943±0.0052	1.9050±0.0303
23	T1-23457	*CWC22*	Pre-mRNA-splicing factor CWC22 homolog	TTCCATTGGTCTTGGTGGCA / GCTGCCGGAACTACCAGATT	3.35	127	82.20	0.9980±0.0021	2.0090±0.0587
24	T3-4203	*PhLP3*	Phosducin-like protein 3	CGGATTTCGTCCGAGAGGTT / AGCAGCAAGCTCCTCTAAGC	3.36	119	84.02	0.9991±0.0003	1.9537±0.0850
25	T3-24436	*B3GALT20*	Probable beta-1,3-galactosyltransferase 20	GCAGAACGCATGGCTGTTAG / AACCTCCTTCCTCGGACTCA	3.45	105	83.29	0.9984±0.0014	1.9437±0.0105
26	T1-26066	*USP13*	Ubiquitin carboxyl-terminal hydrolase 13	GAGTCGTCACGCCTACGAAA / ATGCCACACAGGATGTCGAG	3.47	112	82.24	0.9991±0.0002	1.9333±0.0406
27	T1-29860	*TUBB3*	Tubulin beta-3 chain	CCGTTGTGGAACCATACAATGC / GTAGCCGAGATGAGGTGATTGA	19.52	163	85.18	0.9801±0.0129	1.8890±0.0427
28	/	*EF-1α*	Elongation factor-1α(EF-1α)	GCTTGAGAAGGAGCCCAAGT/CCAACAGCCACAGTTTGTCG	/	150	85.48	0.9994±0.0004	1.9360±0.0070
29	/	*GAPDH*	glyceraldehyde-3-phosphate dehydrogenase	GGCGACTCCCCTCACTACTA/CAGGCATCTCATTGCCCAGA	/	147	81.58	0.9983±0.0017	1.9260±0.0181
30	T3-19020	*Actin1*	Actin	TGAGCGTGAGATTGTGAGAGAC / GATTCCTGCTGCTTCCATACCA	127.68	211	85.23	0.9983±0.0006	1.9610±0.0170
31	T2-31301	*SAND*	SAND	CCTTGCAAAGCAACCAGCAA / GTTGCAGAAGAAGCAGGCAG	207.74	224	86.52	0.9993±0.0006	1.9650±0.0302
32	T1-14202	*Actin85C*	Actin-85C (Fragment)	AGCATTGTTGGTCGTCCTC / TCATCTTTTCCCGATTAGCC	21.24	261	84.68	0.9987±0.0008	2.0467±0.0111
33	T3-18105	*Actin7*	Actin-7	GGTATGGAGGCTGCTGGTA / TGCTGGAATGTGCTCAAGG	51.52	260	84.39	0.9988±0.0004	1.9553±0.0222

* Note

1. The RT-qPCR amplification efficiency (E) and correlation coefficients (R^2^) were determined with LinRegPCR software.

2. S-T was calculated by the formula: (RPKM-MAX—RPKM-MIN)/RPKM-AVERAGE*100. (RPKM represents Reads Per Kilobase Million in transcriptomic analysis.)

3. Amplicon Tm represents the temperature of 50% double-stranded DNA melted to single chain.

4. No.1-31 were tested genes, No.32 and No.33 were 2 members of *Actin* gene family for Validating the expression stability of housekeeping genes.

RT-qPCR was performed using SYBR Green I on LightCycler^®^96 instrument (Roche Diagnostics). The PCR reaction volume was 10μl, which contains 0.5μl diluted cDNA solution, 5μl SYBR Premix Ex Taq^™^ II (Tli RNaseH Plus) (TaKaRa), and 0.8μl each of 5μM primer. The amplification program was set as follows: 30s at 95°C for preincubation, 40 cycles of 5s at 95°C for template denaturation, 10s at 60°C for annealing and 25s at 72°C for extension. Afterwards, a protocol with 10s at 95°C, 60s at 65°C and 1s at 97°C was used for melting curve analysis.

Selection of IRGs was finished with 2 steps. The primary 31 tested genes were first analyzed with 3 technical replicates for selecting candidate IRGs (CIRGs) according to average expression stability (M) values calculated by geNorm with both of protocorms and tissues. CIRGs were then analyzed for selecting IRGs with 3 biological replicates and 3 technical replicates. In technical replicate, cDNA in the RT-qPCR mixture was come from the same cDNA sample, while in biological replicate, cDNA in the RT-qPCR mixture come from different cDNA samples of same type of plant material. The difference of the cycle threshold values (Ct) was set to < 0.25 and the mean was used for RT-qPCR analysis among 3 technical replicates.

### 2.4 Assessment of gene expression stability and determination of IRGs

PCR amplification efficiency of each primer pairs was calculated by LinRegPCR program based on the raw fluorescence data taken from LightCycler^®^96 instrument. 3 application softwares (geNorm, NormFinder and Bestkeeper) were used to estimate gene expression stability of CIRGs under different samples. Raw Ct values from the RT-qPCR experiments conducted on the LightCycler^®^96 instrument were directly used for gene expression stability calculations for Bestkeeper analysis [21], and then transformed to relative quantities by the delta-Ct method for geNorm and NormFinder analysis. RefFinder (a user-friendly web-based comprehensive tool, which integrates the currently available major computational programs geNorm, Normfinder, BestKeeper and the comparative 2^-ΔΔCt^ method) [22] program was also used to estimate the comprehensive ranking of CIRGs based on raw Ct values. geNorm program was also used to determine the optimal number of IRGs required for effective normalization.

### 2.5 Validation of IRGs in protocorm development and temperature stress

*GNOM* had continuous expression in the whole process of embryonic development [23]. *AP2* genes participate in many aspects of plant development [24,25], most of the known functions of *AP2*-*like* genes are important for developmental processes [24,26], for example, *AtAP2* gene has many important functions in reproductive development and seed development [27–29]. In this study, a *GNOM* and *AP2* gene from *D*. *officinale* [30] was used to validate the IRGs in protocorm development. RT-qPCR primer pairs of *GNOM* were designed as that the forward primer is 5’-CTTGTTTTCGGGTTGTTCAT-3’ and the reverse 5’-GTTTGCCATTGCTTTTGCTA-3’. The primer pairs of *AP2* were designed as that the forward is 5’-GAAACCTATCCGCCACAGA-3’ and the reverse 5’-CATCCTAACGAACCCTCCA-3’.

One of *temperature*-*inducedlipocalin* (*TIL*) gene in *D*. *officinale* was cloned and used to demonstrate the usefulness of IRGs in RT-qPCR. RT-qPCR primer pairs of *TIL* were designed as that the forward primer is 5’-AGAGAAAATGGGGAAAGGGAGC-3’ and reverse 5’-CTGGGTTGGAAAAACGAAGGTA-3’.

## Results

### 3.1 Amplification specificity and efficiency of tested genes

In order to guarantee the accuracy of the data for further analysis, the specificity of the primer pairs of 31 tested genes, 2 members of *Actin* gene family and 3 specific genes for validation were detected before RT-qPCR experiment. The melting curve and agarose gel electrophoresis were used to estimate amplification specificity by the presence of a single peak (S1 Fig) and a single band of expected size (S2 Fig) for each primer pairs. No primer dimmers and non-specific amplification were detected in negative control. In positive control, each primer pair performed a specific and expected size of PCR products with the recombinant T vector as DNA template. The melting temperatures (*Tm*) of all PCR products ranged from 80.92 for *KU8* to 86.52 for *SAND* (Table 1). PCR amplification efficiency of each primer pair was between 1.8890±0.0427 for *TUBB3* and 2.0467±0.0111 for *DoActin85C* (Table 1). Correlation coefficients ranged between 0.9801±0.0129 for *TUBB3* and 0.9996±0.0002 for *HDAC5* and *TFIIB* (Table 1). All these results implicate that the primer pairs are adequate for RT-qPCR analysis.

### 3.2 CIRGs’ selection and expression profile analysis

**Fig 1:**
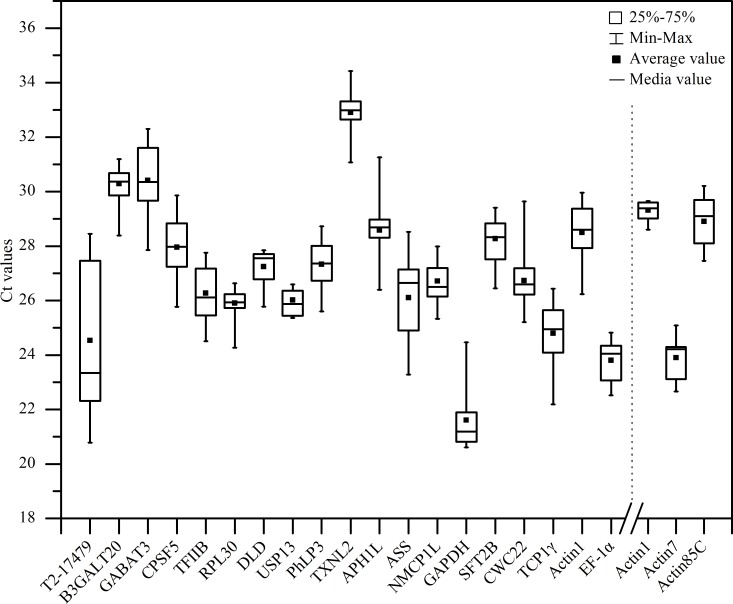
RT-qPCR Ct values of CIRGs across all samples. Lines across the boxes depict the medians. The box indicates the 25th and 75th percentiles, whisker represent the maximum and minimum values. (A) represents 19 CIRGs in all samples; (B) represents 3 members of *Actin* family used for evaluating the expression stability of housekeeping genes in protocorms.

A total of 19 tested genes were selected as CIRGs for normalization of gene expression measures according to M values (S2 Table), in which most of housekeeping genes in this study should be included in CIRGs for comparing the efficiency between novel stably expressed genes from transcriptomic analysis and commonly used housekeeping genes. The cycle threshold (Ct) values were obtained from 19 reactions with 19 RT-qPCR primer pairs of CIRGs and the expression levels of CIRGs across all samples were significantly different (Fig 1). The mean Ct values of the 19 CIRGs ranged from 20.60 to 34.43. Among all CIRGs, *GAPDH* had the lowest Ct values (21.36), indicating it was the most abundant reference transcript, while *TXNL2* was the least abundant transcript with Ct of 33.07. *EF-1α*, *TCP1γ*, and *T2-17479* were moderately expressed. Expression stability was regarded as the most important characteristics for reference genes, it can be detected by calculating the coefficient of variance (CV) of Ct values. *T2-17479* showed the largest gene expression variation, indicating that the gene was unstable, while *RPL30*, *DLD*, *USP13*, *NMCP1L*, *EF-1α* and *Actin7* had narrow expression range (less than 2.67) in their total sample.

### 3.3 Expression stability analysis of CIRGs

**Fig 2:**
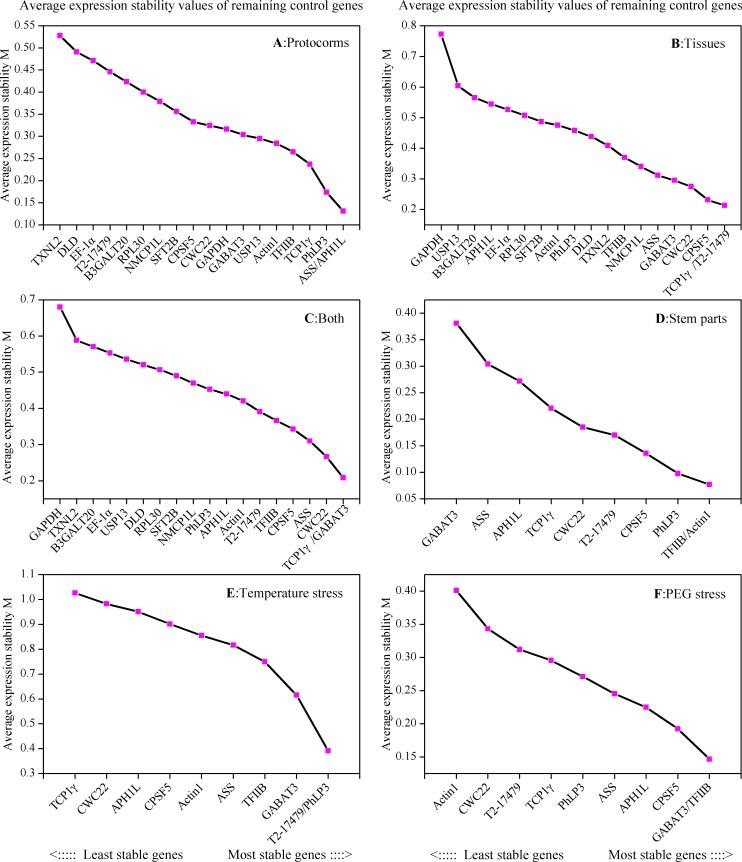
Average expression stability values (M) calculated by geNorm. Expression stability and ranking of 19 CIRGs in (A) different development stages of protocorm, (B) different tissues, (C) both of protocorms and tissues, (D) stem parts, (E) temperature stressed, (F) PEG stressed.

Expression stability of CIRGs were calculated by geNorm based on the M values [31] and the cut-off value was set as 1.5. The most stable reference gene has the lowest M value, on the contrary, highest M value showed reference gene the least stable. In our study, M values of CIRGs were plotted in Fig 2 and all of them less than 1.5, which showed that all CIRGs were rather stable. In protocorms, *ASS* and *APH1L* had the highest expression stability (the lowest M values), whereas *TXNL2* was revealed the least stability (Fig 2A). In different tissues, *TCP1γ* and *T2-17479* were the most stable genes, while *GAPDH* was the least stable gene (Fig 2B). If the protocorms and tissues (labeled as both) were taken together, the most stably expressed genes were *TCP1γ* and *GABAT3* (Fig 2C). The first 10 CIRGs from both of protocorms and tissues were selected for further study in stem parts and stressed plantlets. *TFIIB* and *Actin1* performed the most stable expression in stem parts (Fig 2D), while in temperature stress, *T2-17479* and *PhLP3* were the most stable genes (Fig 2E), and in PEG stress, the most stable genes were *GABAT3* and *TFIIB* (Fig 2F).

The stability value and rank of CIRGs calculated by NormFinder was showed in Tables 2 and 3. The results showed that *ASS* had the least stability value in protocorms, both and PEG stressed samples, indicating it was the most stable IRG in these samples. In different tissues and stem parts, the most stable reference genes were *T2-17479*. *TFIIB* showed the most expression stability under temperature stress. However, the ranking was varied from different samples, for example, *ASS* had the opposite result in stem parts and temperature stressed plantlets. Besides, housekeeping genes *GAPDH*, *EF-1α* and *Actin1* were not a good choice with their higher expression stability values.

**Table 2:** Rank of 19 CIRGs calculated by NormFinder and BestKeeper.

Rank	NortmFinder			BestKeeper		
	Protocorms	Tissues	Both	Protocorms	Tissues	Both
1	*ASS* (0.020)	*T2-17479* (0.034)	*ASS* (0.074)	*APH1L*(0.68±0.20)	*USP13*(1.44±0.37)	*T2-17479*(1.23±0.34)
2	*APH1L* (0.073)	*ASS* (0.055)	*CPSF5* (0.079)	*ASS*(0.69±0.17)	*PhLP3*(1.45±0.40)	*GABAT3*(1.37±0.43)
3	*TFIIB* (0.080)	*TXNL2* (0.065)	*TCP1γ* (0.081)	*GABAT3*(0.79±0.25)	*T2-17479*(1.52±0.42)	*CPSF5*(1.45±0.42)
4	*PhLP3* (0.098)	*PhLP3* (0.086)	*TFIIB* (0.085)	*TCP1γ*(0.86±0.22)	*ASS*(1.60±0.38)	*Actin1*(1.46±0.43)
5	*USP13* (0.100)	*CPSF5* (0.097)	*PhLP3* (0.085)	*PhLP3*(0.98±0.28)	*CWC22*(1.69±0.45)	*TCP1γ*(1.47±0.37)
6	*TCP1γ* (0.101)	*TCP1γ* (0.112)	*Actin1* (0.096)	*Actin1*(0.99±0.29)	*TXNL2*(1.73±0.57)	*APH1L*(1.50±0.42)
7	*Actin1* (0.125)	*TFIIB* (0.118)	*SFT2B* (0.098)	*CPSF5*(1.12±0.32)	*EF-1α*(1.79±0.42)	*USP13*(1.57±0.41)
8	*SFT2B* (0.138)	*NMCP1L* (0.119)	*NMCP1L* (0.099)	*RPL30*(1.14±0.30)	*GABAT3*(1.87±0.58)	*CWC22*(1.65±0.45)
9	*CPSF5* (0.142)	*Actin1* (0.120)	*T2-17479* (0.113)	*B3GALT20*(1.17±0.35)	*Actin1*(1.88±0.55)	*RPL30*(1.67±0.43)
10	*CWC22* (0.149)	*DLD* (0.126)	*RPL30* (0.118)	*T2-17479*(1.18±0.33)	*TCP1γ*(2.03±0.51)	*TFIIB*(1.69±0.46)
11	*GAPDH* (0.150)	*RPL30* (0.145)	*B3GALT20* (0.127)	*TFIIB*(1.36±0.37)	*CPSF5*(2.04±0.58)	*ASS*(1.71±0.42)
12	*GABAT3* (0.152)	*SFT2B* (0.149)	*CWC22* (0.141)	*DLD*(1.53±0.42)	*DLD*(2.15±0.58)	*PhLP3*(1.78±0.50)
13	*NMCP1L* (0.184)	*EF-1α* (0.151)	*GABAT3* (0.142)	*USP13*(1.53±0.40)	*TFIIB*(2.17±0.59)	*B3GALT20*(1.87±0.57)
14	*RPL30* (0.187)	*B3GALT20* (0.152)	*DLD* (0.157)	*CWC22*(1.58±0.43)	*APH1L*(2.23±0.62)	*DLD*(1.93±0.53)
15	*TXNL2* (0.199)	*CWC22* (0.172)	*APH1L* (0.164)	*EF-1α*(1.67±0.40)	*RPL30*(2.30±0.59)	*TXNL2*(1.97±0.65)
16	*B3GALT20* (0.206)	*GABAT3* (0.197)	*EF-1α* (0.177)	*NMCP1L*(1.70±0.46)	*NMCP1L*(2.38±0.63)	*SFT2B*(2.13±0.60)
17	*EF-1α* (0.236)	*APH1L* (0.253)	*TXNL2* (0.189)	*SFT2B*(1.93±0.55)	*SFT2B*(2.40±0.67)	*NMCP1L*(2.16±0.58)
18	*DLD* (0.249)	*USP13* (0.356)	*USP13* (0.234)	*TXNL2*(1.95±0.65)	*B3GALT20*(2.86±0.87)	*EF-1α*(2.71±0.65)
19	*T2-17479* (0.262)	*GAPDH* (0.606)	*GAPDH* (0.388)	*GAPDH*(1.98±0.43)	*GAPDH*(5.38±1.17)	*GAPDH*(3.34±0.72)

**Table 3:** Rank of the more stable 10 CIRGs by NormFinder and BestKeeper.

Rank	NortmFinder			BestKeeper		
	Stem parts	Temperature stress	PEG stress	Stem parts	Temperature stress	PEG stress
1	*T2-17479* (0.029)	*TFIIB* (0.141)	*ASS* (0.051)	*T2-17479*(0.70±0.15)	*Actin1*(1.65±0.47)	*GABAT3*(0.53±0.16)
2	*PhLP3* (0.053)	*PhLP3* (0.176)	*TFIIB* (0.059)	*TCP1γ*(0.71±0.17)	*GABAT3*(1.78±0.53)	*TFIIB*(0.77±0.20)
3	*CPSF5* (0.062)	*CWC22* (0.180)	*CPSF5* (0.061)	*CWC22*(0.84±0.22)	*PhLP3*(1.79±0.48)	*APH1L*(0.81±0.23)
4	*Actin1* (0.063)	*GABAT3* (0.205)	*TCP1γ* (0.097)	*Actin1*(0.90±0.24)	*ASS*(2.13±0.59)	*CPSF5*(0.82±0.23)
5	*TFIIB* (0.084)	*Actin1* (0.210)	*GABAT3* (0.131)	*GABAT3*(1.02±0.29)	*APH1L*(2.21±0.65)	*ASS*(1.29±0.35)
6	*CWC22* (0.087)	*APH1L* (0.217)	*T2-17479* (0.140)	*CPSF5*(1.11±0.29)	*TFIIB*(2.34±0.61)	*PhLP3*(1.31±0.35)
7	*TCP1γ* (0.155)	*ASS* (0.225)	*PhLP3* (0.147)	*PhLP3*(1.18±0.31)	*CPSF5*(2.59±0.72)	*Actin1*(1.34±0.38)
8	*ASS* (0.200)	*T2-17479* (0.233)	*CWC22* (0.148)	*TFIIB*(1.20±0.30)	*CWC22*(2.86±0.77)	*CWC22*(1.39±0.37)
9	*APH1L* (0.203)	*TCP1γ* (0.263)	*APH1L* (0.152)	*APH1L*(1.68±0.45)	*T2-17479*(2.88±0.65)	*TCP1γ*(1.77±0.43)
10	*GABAT3* (0.355)	*CPSF5* (0.281)	*Actin1* (0.247)	*ASS* (2.03±0.53)	*TCP1γ*(3.72±0.91)	*T2-17479*(2.01±0.46)

Based on the coefficient of variance (CV) and the standard deviation (SD) of the Ct values [32,33], the results of BestKeeper analysis were also showed in Tables 2 and 3. It showed that *APH1L*, *USP13* and *T2-17479* had CV±SD values of 0.68±0.20, 1.44±0.37 and 1.23±0.34 in protocorms, tissues and both of protocorms and tissues, indicating they were the most stable genes. In stem parts, the most stable gene was the same as the both samples. While under abiotic stress of temperature and PEG, the most stable genes were *Actin1* and *GABAT3*, with the lowest CV±SD values.

**Fig 3:**
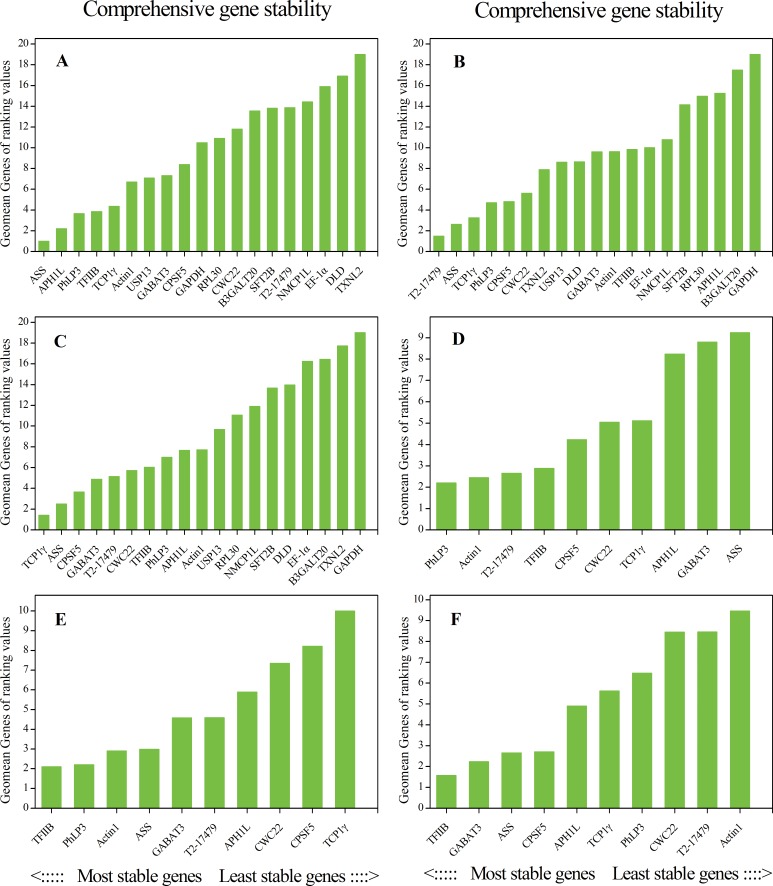
Comprehensive ranking of CIRGs in all samples, according to their expression stability values as given by RefFinder based on geNorm, NormFinder, BestKeeper, and comparative delta-Ct methods. A~F represents protocorms, tissues, both of protocorms and tissues, stem parts, temperature stress and PEG stress respectively.

The comprehensive gene stability calculated by RefFinder was showed in Fig 3. In protocorms, *ASS* was the stablest IRG, *TXNL2* was the unstablest IRG; in tissues *T2-17479* was the stablest IRG, *GAPDH* was the unstablest IRG (Fig 3A and 3B), While taking both protocorms and tissues together, the rank of CIRGs was different with protocorms and tissues, the stablest IRG was *TCP1γ*, the unstablest IRG was same as tissues (Fig 3C). In stem parts, *PhLP3* ranked at the top of CIRGs, while *ASS* showed the least expression stability (Fig 3D). *TFIIB* was the stablest IRG in both temperature and PEG stressed, *TCP1γ* and *Actin1* were the unstablest IRGs respectively (Fig 3E and 3F).

### 3.4 Determination of optimal IRGs

**Fig 4:**
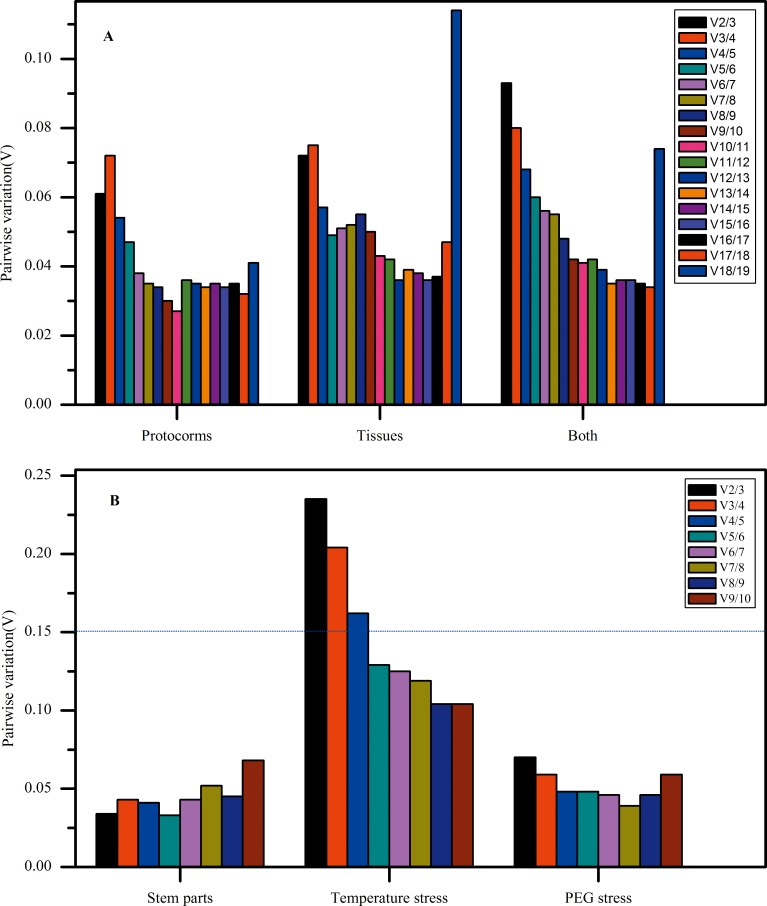
Determination of the optimal number of IRGs required for effective normalization. Pairwise variations Vn/Vn+1 was analyzed between the normalization factors NFn and NFn+1 to determine the minimum number of IRG required for qPCR data normalization in various samples. (A) Pairwise variation of 19 CIRGs in protocorms, tissues and both of protocorms and tissues. (B) Pairwise variation of 10 CIRGs in stem parts, temperature (cold, room and heat) and PEG stressed plantlets.

The geNorm also performs stepwise calculations of the pair-wise variation (Vn/Vn+1) between sequential normalization factors (NFn and NFn+1) to determine the optimal number of reference genes required for accurate normalization [34], and the cut-off value was usually set as 0.15. The larger pairwise variation means that the added gene had a more significant effect and should preferably be included to calculate a reliable normalization factor [1]. The pairwise variations (V) across all samples (Fig 4) were all less than the cut-off value (0.15) except the temperature stressed samples, indicating that two stable IRG were enough to normalize gene expression. When the samples were under temperature stressed, the pairwise variation of V2/3, V3/4 and V4/5 was 0.235, 0.204, 0.162 respectively (Fig 4B), higher than 0.15, indicating that they were necessary to add the fifth CIRG for normalization of gene expression. As was shown in Fig 4, the pairwise variation of V5/6 was 0.129. Thus, at least five CIRG should be included to normalize gene expression under temperature stressed.

### 3.5 Validation of expression stability of housekeeping genes

In the former analyses of CIRGs, 3 housekeeping genes, *Actin1*, *GAPDH* and *EF-1α*, showed worse expression stability than some other CIRGs and were ranked behind. *Actin1* performed more expression stability than other two housekeeping genes. *Actin1* and other two members of *Actin* family (*Actin7* and *Actin85C*) were selected to check the influence and validate the expression stability of housekeeping genes in IRGs’ selection. Expression profile of 3 *Actin* genes in protocorm was showed in Fig 1B. The mean Ct values ranged from 22.66 to 30.21, in which, *Actin7* had the lowest Ct values (24.22), indicating it was the most abundant reference transcript, while *Actin1* was the least abundant transcript with Ct of 29.38. Although *Actin1* had the highest Ct value among 3 *Actin* genes, it had the narrowest expression range (1.06) less than the 2.42 of *Actin7* and 2.75 of *Actin85C*, which also indicated that *Actin1* could be more stable on the other hand. Rank and expression stability value of 3 *Actin* genes in protocorms was showed in Tables 4 and S3, it can be seen that *Actin1* was stabler than *Actin7* and *Actin85C*, which was consistent with the analysis of expression profile, indicating that the primary screen of commonly used housekeeping genes as CIRGs was persuasive and the other members of a housekeeping gene were not better than the commonly used ones. In extreme condition, such as under temperature stress, the optimal number of IRGs was five genes’ combination according to geNorm analysis (Fig 4B), which was enough to normalize the interested genes. However, in some cases, such as in stem parts, *Actin1* was a better reference gene for expression normalization; while in protocorms, the stablest *Actin1*among 3 *Actin* genes only ranked at 5, thus it was not essential that *Actin1* could be included in optimal IRGs for normalization of interesting genes. In summary, the housekeeping genes, such as *Actin1*, were not suitable for gene expression normalization in all kinds of biological processes, optimal IRG or IRGs should be determined according to the specific experiment condition.

**Table 4:** Validation of expression stability of housekeeping genes.

Gene	Rank			
	geNorm	NormFinder	BestKeeper	RefFinder
*Actin1*	5 (0.284)	9(0.140)	6(0.291±0.993)	7(7.14)
*Actin7*	18 (0.514)	15(0.201)	19(0.640±2.677)	19(19.00)
*Actin85C*	19 (0.546)	19(0.228)	21(0.786±2.719)	20(20.25)

### 3.6 Validation of IRGs with *GNOM*, *AP2* in protocorm development and *TIL* in temperature stressed process

**Fig 5:**
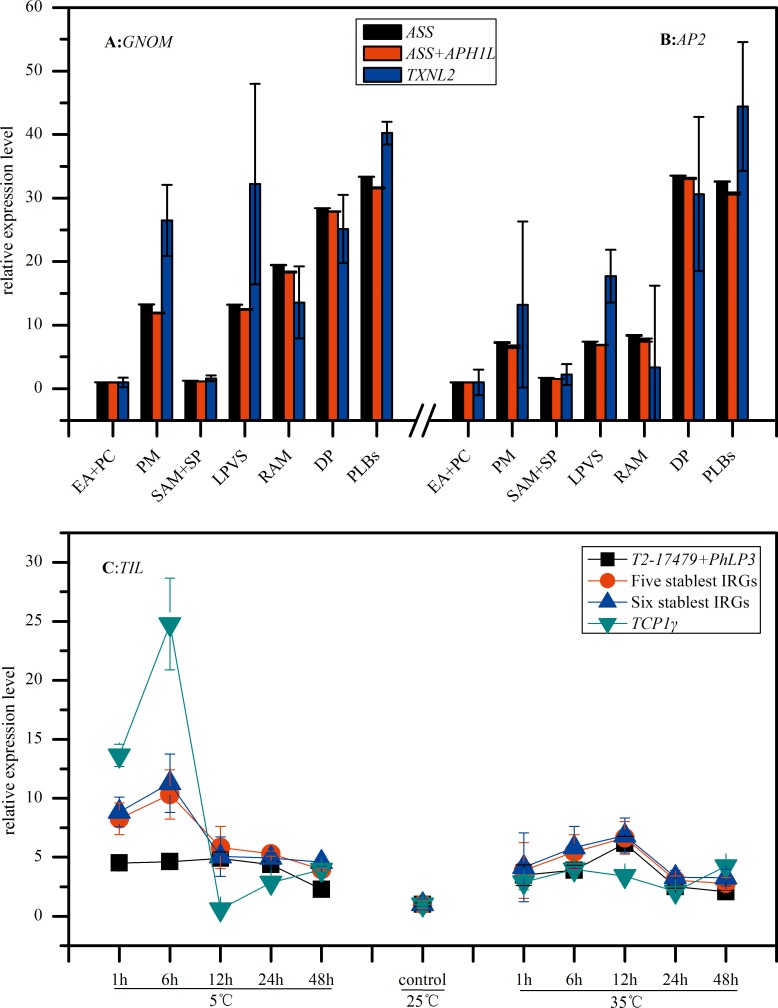
Relative quantification of *GNOM*, *AP2* and *TIL* expression using validated reference genes for normalization in protocorm and temperature stress. (A, B) *ASS* was the stablest gene, *ASS*+*APH1L* was the best combination reference genes. *TXNL2* was the unstablest gene. (C) *T2-17479*+*PhLP3* was the best combination reference genes; *T2-17479*, *PhLP3*, *GABAT3*, *TFIIB* and *ASS* were the optimal 5 stablest IRGs; *T2-17479*, *PhLP3*, *GABAT3*, *TFIIB*, *ASS* and *Actin1* were the 6 stablest IRGs. All these IRGs above were calculated by geNorm.

It has been documented that the use of inappropriate reference genes can dramatically change the interpretation of the expression pattern of a given target gene [35]. For protocorms, 2 IRGs are enough for effective normalization (Fig 4A), and 2 stablest IRGs are *ASS* and *APHIL* from 4 application softwares. The relative expression level of *GNOM*, *AP2* normalized by different reference genes were showed in Fig 5A and 5B. The difference between expression levels normalized by 1 and 2 stablest reference genes was not significant, while it is significant between 1 or 2 stablest reference genes and the unstablest one (*TXNL2*). *TIL* gene expression under temperature stress was showed in Fig 5C. Its expression level in cold/heat stress was many times higher than in room temperature, and it more violently fluctuated in cold stress (5°C). For gene expression normalization, it needed at least 5 stablest IRGs, which were *T2-17479*, *PhLP3*, *GABAT3*, *TFIIB* and *ASS*. It was obviously clear that the relative expression level of *TIL* had significantly difference when using adverse reference genes for normalization and adequate IRGs and number of IRGs are very important for gene expression normalization. Using the unstablest IRG (*TCP1γ*) led to large difference in tendency and relative expression level of *TIL*. Using the stablest IRGs led to similar tendency, but significantly different relative expression level. There were significant difference in relative expression level between 2 stablest IRGs (*T2-17479*, *PhLP3*) and 5 stablest IRGs, while no significant difference between 5 stablest IRGs and 6 stablest IRGs (*T2-17479*, *PhLP3*, *GABAT3*, *TFIIB*, *ASS* and *Actin1*), it indicated that it’s not essential for gene expression normalization with more than optimal number of IRGs.

## Discussion

Because of its health benefit and its slow growth cycle, wild *D*. *officinale* like many other *Dendrobium* species, has been exploited to near extinction and is now classified as one of the rare and endangered medicinal plants of China [30,36]. Studies on *D*.*officinale* had a long history and had increased many researchers’ interest in recent years for its health and medical function as well as the ornamental value. However, the molecular regulatory mechanism on growth, especially on protocorm development, at the genomic level remains unclear. In order to find out the expression profile of some specific genes on protocorm development, it was necessary to select and validate the effective IRGs for normalization of some specific genes.

Expression level was an important index in studying the molecular mechanism of some specific genes in regulating the plant development, thus some means were developed to accurately measure the expression level of interested genes, especially for the relative expression level among multiple genes. Semiquantitation and RT-qPCR were the commonly used means for expression normalization. In pregenomic era, housekeeping genes were most commonly considered as reference genes in basic cellular processes [37], such as *Actin*, *SAND*, *Tubulin*, *ubiquitin* and so on. Nevertheless, numerous researchers have already shown that the expression of these traditional genes might also be variational [12,38–40], this could also be seen from the expression stability analysis of 5 housekeeping genes in this study. Thus, normalization with multiple reference genes is becoming popular and standard in plant research [33,41].

It was surprising that the most popular housekeeping genes, *Actin1*, *Actin7 and Actin85C*, *TUBB3*, *GAPDH*, *EF-1α* and *SAND*, performed poorly as reference genes in this study. From the analysis above, *TUBB3* and *SAND* were the 2 unstablest reference genes among 5 tested housekeeping genes in multiple samples of different biological processes. In protocorms, tissues and both, *Actin1* showed more expression stability than *GAPDH* and *EF-1α*. In tissues and both samples, *GAPDH* showed the highest stability values. All these results reflected these commonly used housekeeping genes were variable, which also confirmed the importance to select appropriate reference genes for normalization of gene expression.

For further calculating the influences of different members of housekeeping gene family, three members of *Actin* family in *D*. *officinale* were selected according to the expression difference in transcriptome for RT-qPCR experiment. In analysis above, three *Actin* genes ranked at the back of the line among all samples calculated by 4 application softwares, except *Actin1* in stem parts. The commonly used *Actin1* had more expression stability than *Actin7* and *Actin85C*, but it only ranked at 6, 11, 10, 2, 3 and 10 successively calculated by RefFinder (Fig 3) across the 6 kinds of samples in this study, being inferior to the other CIRGs from the transcriptome. So, some housekeeping genes were not suitable as effective IRGs because of their low expression stability in a given biological process,

At present, RT-qPCR has significantly improved the detection and quantification of expression profiles of target genes in distinct biological processes, especially for the lower abundant genes. The main advantages of this technique are its high sensitivity, high specificity and broad quantification range [42,43]. For these reasons, RT-qPCR is the first choice for accurate and sensitive quantification of gene expression profiles. As gene expression level was becoming a research hotspot, it is necessary to screen the internal control genes for gene expression normalization. An ideal control gene or IRG should be relatively stably expressed in different development stages, different tissues and some other samples exposed to different experiment conditions. But actually, it’s not always the cases, such as *ASS* was the stablest IRG in protocorms, but not in stem parts; *Actin1* stabler in stem parts, but not in protocorms, and so on. So, there may be no universal IRG or IRGs suitable for all biological processes for gene expression normalization. An effective mean should be used for finding the optimal IRG or IRGs in a given biological process. And as long as the IRG or IRGs were determined, it could be used for almost all genes’ expression normalization in this process. It was only possible to screen the best IRG or IRGs from the high-throughput level, such as whole transcriptomic analysis in our study and high-density oligonucleotide array-based expression profiles analysis [41], in a biological process. So, the appropriate IRGs could be selected form the whole transcriptomic analysis.

Although most authors agree in using only one single gene as an internal control for normalization, it has been suggested that using two or more reference genes for RT-qPCR studies might generate more reliable results [1,44]. In this study, 1 stablest IRG or 2 stablest IRGs were identical by 4 application softwares in protocorms and the relative expression level of interested genes normalized by this or these IRGs showed no significant difference (Fig 5A and 5B), indicating that if IRG or IRGs was appropriate, number of IRGs will not significantly influence the result of interested gene expression normalization. So, a few IRGs, even 1~2 IRGs were enough to used as the internal control reference genes for other genes normalizations in a specific biological process. On contrary, using inappropriate IRGs will lead to large difference (Fig 5C), and a certain number of optimal IRGs should be included to ensure the accuracy of the normalization of genes expression.

To evaluate the best IRG or IRGs for protocorms, tissues, both of protocorms and tissues, stem parts, temperature stress and PEG stress in *D*. *officinale*, three different statistical approaches, geNorm, NormFinder and BestKeeper, were utilized to identify the expression stability of 19 CIRGs. The most prominent observation was that each type of software produced a different set of top-ranked reference genes, since they are based on distinct statistical algorithms [34]. In protocorms, 2 stablest IRGs were *ASS* and *APH1L* according to 3 application softwares and rank of 19 CIRGs showed least difference; however, the top stablest IRGs and rank of 19 CIRGs showed greater difference in tissues and other samples. It indicated that not only the statistical algorithms, but origin of CIRGs would influence the top stablest IRGs and rank of CIRGs. So, it is best to identify IRG or IRGs from the stably expressed genes in a given biological process and there were no greater difference among application softwares if CIRGs come from high-throughout sequence data.

## Conclusions

In this report, using most stably expressed genes from whole transcriptome as CIRGs and some application softwares, such as geNorm, *ASS* and *APH1L* were determined as the optimal IRGs for gene expression normalization in protocorm development of *D*. *officinale*. This work will be very useful for further gene expression analysis and finding the regulatory mechanism of protocorm development.

## Supporting Information

S1 Fig Gene specificity. Melting curves of 31 tested genes, other 2 members (*Actin85C* and *Actin7*) of *Actin* gene family and 3 sepecific genes (*GNOM*, *AP2*, *TIL*) for validating the usefulness of IRG or IRGs, all these genes show single peaks.
(TIF)

S2 Fig Agarose Gel analysis of RT-qPCR amplicon product. The lanes of 1–33 represent the CIRGs and their order were the same as the No. listed in Table 1. The lanes of 34–36 represent *TIL*, *GNOM* and *AP2*, respectively.
(TIF)

S1 Table The RPKM of 31 tested genes in transcriptome. (DOCX)

S2 Table The primary evaluation of expression stability values of 31 tested genes. (DOCX)

S3 Table Validation of expression stability of housekeeping genes. (DOCX)
