# Supplementary figures and images for: Whole-Transcriptome Selection and Evaluation of Internal Reference Genes for Expression Analysis in Protocorm Development of Dendrobium officinale Kimura et Migo
Source: PLoS One. 2016 Nov 4;11(11):e0163478. doi: 10.1371/journal.pone.0163478 (PMC5096709; doi:10.1371/journal.pone.0163478)

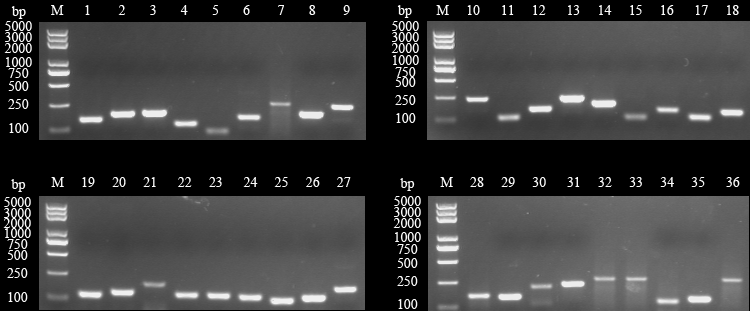

Supplement: S2 Fig — The lanes of 1–33 represent the CIRGs and their order were the same as the No. listed in Table 1. The lanes of 34–36 represent TIL, GNOM and AP2, respectively. (TIF) [file pone.0163478.s002.tif]
