# Supplementary material for: Whole-Transcriptome Selection and Evaluation of Internal Reference Genes for Expression Analysis in Protocorm Development of Dendrobium officinale Kimura et Migo
Source: PLoS One. 2016 Nov 4;11(11):e0163478. doi: 10.1371/journal.pone.0163478 (PMC5096709; doi:10.1371/journal.pone.0163478)
Supplement: S1 Table — (DOCX) [file pone.0163478.s003.docx]

**S1 Table. The RPKM of 31 tested genes in transcriptome**

| No. | Unigene No. | Gene Symbol | T3-RPKM | T1-RPKM | T2-RPKM |
| --- | --- | --- | --- | --- | --- |
| 1 | T2-29401 | *GABAT3* | 46.28011 | 46.56072 | 46.23186 |
| 2 | T2-34503 | *NMCP1L* | 22.38165 | 22.53175 | 22.27494 |
| 3 | T2-26423 | *MOS2* | 27.62528 | 27.49767 | 27.93966 |
| 4 | T2-29783 | *PAXIP1* | 9.85009 | 9.687523 | 9.837109 |
| 5 | T3-26893 | *HDAC5* | 14.73979 | 14.6957 | 14.9455 |
| 6 | T1-28366 | *KU8* | 14.52454 | 14.50713 | 14.75417 |
| 7 | T1-23605 | *RPL30* | 136.3597 | 136.9876 | 138.9656 |
| 8 | T3-27882 | *SFT2B* | 8.220884 | 8.408678 | 8.271461 |
| 9 | T2-34737 | *UBC24* | 40.22656 | 40.61519 | 41.18094 |
| 10 | T2-17479 | *T2-17479* | 211.1168 | 209.8983 | 205.8367 |
| 11 | T3-12872 | *TPRXL* | 12.40961 | 12.71517 | 12.72932 |
| 12 | T3-20348 | *CPSF5* | 9.746145 | 9.997557 | 9.816564 |
| 13 | T3-17931 | *TFIIB* | 54.43124 | 53.21117 | 54.61571 |
| 14 | T2-29412 | *APH1L* | 21.19613 | 21.53463 | 20.94413 |
| 15 | T3-22550 | *BIP1* | 15.03886 | 14.99084 | 14.60961 |
| 16 | T3-13226 | *GT3b* | 1.910382 | 1.875861 | 1.855854 |
| 17 | T3-11823 | *T3-11823* | 2.233301 | 2.192945 | 2.169557 |
| 18 | T3-12831 | *T3-12831* | 8.523007 | 8.270535 | 8.349316 |
| 19 | T1-29649 | *ASS* | 103.7581 | 100.7404 | 100.6738 |
| 20 | T1-22498 | *TCP1γ* | 97.12319 | 98.62341 | 100.1163 |
| 21 | T3-23608 | *DLD* | 20.00739 | 19.63171 | 20.24593 |
| 22 | T2-21987 | *TXNL2* | 9.806912 | 9.501303 | 9.690317 |
| 23 | T1-23457 | *CWC22* | 20.92578 | 20.24096 | 20.93532 |
| 24 | T3-4203 | *PhLP3* | 46.21337 | 46.90061 | 47.79205 |
| 25 | T3-24436 | *B3GALT20* | 9.538569 | 9.802116 | 9.471121 |
| 26 | T1-26066 | *USP13* | 38.64787 | 37.33393 | 37.7144 |
| 27 | T1-29860 | *TUBB3* | 469.1236 | 477.6147 | 567.662 |
| 28 | / | *EF-1α* | - | - | - |
| 29 | / | *GAPDH* | - | - | - |
| 30 | T3-19020 | *Actin1* | 16.0168 | 36.8389 | 67.04464 |
| 31 | T2-31301 | *SAND* | 0.979388 | 4.487887 | 15.49478 |
| 32 | T1-14202 | *Actin85C* | 7.514402 | 6.054248 | 7.059265 |
| 33 | T3-18105 | *Actin7* | 1013.079 | 575.7627 | 957.778 |
