# Supplementary material for: Whole-Transcriptome Selection and Evaluation of Internal Reference Genes for Expression Analysis in Protocorm Development of Dendrobium officinale Kimura et Migo
Source: PLoS One. 2016 Nov 4;11(11):e0163478. doi: 10.1371/journal.pone.0163478 (PMC5096709; doi:10.1371/journal.pone.0163478)
Supplement: S2 Table — (DOCX) [file pone.0163478.s004.docx]

**S2 Table. The primary evaluation of expression stability values of 31 tested genes**

| No. | Unigene No. | Gene Symbol | M value |
| --- | --- | --- | --- |
| 1 | T2-17479 | *T2-17479* | - |
| 2 | T3-24436 | *B3GALT20* | 0.321 |
| 3 | T2-29401 | *GABAT3* | 0.380 |
| 4 | T3-20348 | *CPSF5* | 0.483 |
| 5 | T3-17931 | *TFIIB* | 0.515 |
| 6 | T1-23605 | *RPL30* | 0.555 |
| 7 | T3-23608 | *DLD* | 0.576 |
| 8 | T1-26066 | *USP13* | 0.591 |
| 9 | T3-4203 | *PhLP3* | 0.599 |
| 10 | T2-21987 | *TXNL2* | 0.609 |
| 11 | T2-29412 | *APH1L* | 0.619 |
| 12 | T1-29649 | *ASS* | 0.632 |
| 13 | T2-34503 | *NMCP1L* | 0.642 |
| 14 | / | *GAPDH* | 0.651 |
| 15 | T3-27882 | *SFT2B* | 0.657 |
| 16 | T1-23457 | *CWC22* | 0.663 |
| 17 | T1-22498 | *TCP1γ* | 0.671 |
| 18 | T3-19020 | *Actin1* | 0.680 |
| 19 | / | *EF-1α* | 0.690 |
| 20 | T3-11823 | *T3-11823* | 0.708 |
| 21 | T2-26423 | *MOS2* | 0.723 |
| 22 | T2-29783 | *PAXIP1* | 0.741 |
| 23 | T2-34737 | *UBC24* | 0.757 |
| 24 | T3-22550 | *BIP1* | 0.772 |
| 25 | T3-12831 | *T3-12831* | 0.785 |
| 26 | T2-31301 | *SAND* | 0.816 |
| 27 | T3-12872 | *TPRXL* | 0.852 |
| 28 | T3-13226 | *GT3b* | 0.887 |
| 29 | T1-28366 | *KU8* | 0.920 |
| 30 | T3-26893 | *HDAC5* | 0.987 |
| 31 | T1-29860 | *TUBB3* | 1.056 |
