# Supplementary material for: Whole-Transcriptome Selection and Evaluation of Internal Reference Genes for Expression Analysis in Protocorm Development of Dendrobium officinale Kimura et Migo
Source: PLoS One. 2016 Nov 4;11(11):e0163478. doi: 10.1371/journal.pone.0163478 (PMC5096709; doi:10.1371/journal.pone.0163478)
Supplement: S3 Table — (DOCX) [file pone.0163478.s005.docx]

**S3 Table. Validation of expression stability of housekeeping genes**

| Rank | geNorm | NormFinder | BestKeeper |
| --- | --- | --- | --- |
| 1 | *ASS*+*APH1L* (0.131) | *ASS* (0.041) | *APH1L* (0.683±0.196) |
| 2 | *PhLP3* (0.174) | *TFIIB* (0.070) | *ASS* (0.685±0.170) |
| 3 | *TCP1γ* (0.237) | *APH1L* (0.079) | *GABAT3* (0.792±0.252) |
| 4 | *TFIIB* (0.265) | *PhLP3* (0.081) | *TCP1γ* (0.855±0.220) |
| 5 | *Actin1* (0.284) | *USP13* (0.100) | *PhLP3* (0.985±0.280) |
| 6 | *USP13* (0.295) | *TCP1γ* (0.122) | *Actin1* (0.993±0.291) |
| 7 | *GABAT3* (0.304) | *SFT2B* (0.131) | *CPSF5* (1.125±0.325) |
| 8 | *GAPDH* (0.317) | *CPSF5* (0.135) | *RPL30* (1.139±0.296) |
| 9 | *CWC22* (0.325) | *Actin1* (0.140) | *B3GALT20* (1.169±0.354) |
| 10 | *CPSF5* (0.333) | *GAPDH* (0.147) | *T2-17479* (1.176±0.326) |
| 11 | *SFT2B* (0.356) | *CWC22* (0.157) | *TFIIB* (1.363±0.370) |
| 12 | *NMCP1L* (0.379) | *NMCP1L* (0.166) | *DLD* (1.526±0.419) |
| 13 | *RPL30* (0.400) | *GABAT3* (0.169) | *USP13* (1.532±0.401) |
| 14 | *B3GALT20* (0.424) | *RPL30* (0.180) | *CWC22* (1.578±0.430) |
| 15 | *T2-17479* (0.445) | *Actin7* (0.201) | *EF-1α* (1.667±0.403) |
| 16 | *EF-1α* (0.470) | *TXNL2* (0.207) | *NMCP1L* (1.703±0.459) |
| 17 | *DLD* (0.490) | *B3GALT20* (0.218) | *SFT2B* (1.935±0.554) |
| 18 | *Actin7* (0.514) | *EF-1α* (0.219) | *TXNL2* (1.951±0.645) |
| 19 | *Actin85C* (0.546) | *Actin85C* (0.228) | *GAPDH* (1.981±0.425) |
| 20 | *TXNL2* (0.576) | *DLD* (0.234) | *Actin7* (2.677±0.640) |
| 21 |  | *T2-17479* (0.273) | *Actin85C* (2.719±0.786) |
